# Spontaneous brain network activity: Analysis of its temporal complexity

**DOI:** 10.1162/NETN_a_00006

**Published:** 2017-06-01

**Authors:** Mangor Pedersen, Amir Omidvarnia, Jennifer M. Walz, Andrew Zalesky, Graeme D. Jackson

**Affiliations:** The Florey Institute of Neuroscience and Mental Health, The University of Melbourne, Melbourne, Victoria, Australia; Department of Psychiatry, Melbourne Neuropsychiatry Centre, The University of Melbourne, Victoria, Australia; Melbourne School of Engineering, The University of Melbourne, Victoria, Australia; Department of Neurology, Austin Health, Melbourne, Victoria, Australia

**Keywords:** Instantaneous phase synchrony, Sample entropy, Brain networks, fMRI, Graph theory

## Abstract

The brain operates in a complex way. The temporal complexity underlying macroscopic and spontaneous brain network activity is still to be understood. In this study, we explored the brain’s complexity by combining functional connectivity, graph theory, and entropy analyses in 25 healthy people using task-free functional magnetic resonance imaging. We calculated the pairwise instantaneous phase synchrony between 8,192 brain nodes for a total of 200 time points. This resulted in graphs for which time series of clustering coefficients (the “cliquiness” of a node) and participation coefficients (the between-module connectivity of a node) were estimated. For these two network metrics, sample entropy was calculated. The procedure produced a number of results: (1) Entropy is higher for the participation coefficient than for the clustering coefficient. (2) The average clustering coefficient is negatively related to its associated entropy, whereas the average participation coefficient is positively related to its associated entropy. (3) The level of entropy is network-specific to the participation coefficient, but not to the clustering coefficient. High entropy for the participation coefficient was observed in the default-mode, visual, and motor networks. These results were further validated using an independent replication dataset. Our work confirms that brain networks are temporally complex. Entropy is a good candidate metric to explore temporal network alterations in diseases with paroxysmal brain disruptions, including schizophrenia and epilepsy.

## Author Summary

In recent years, connectomics has provided significant insights into the topological complexity of brain networks. However, the temporal complexity of brain networks still remains somewhat poorly understood. In this study we used entropy analysis to demonstrate that the properties of network segregation (the clustering coefficient) and integration (the participation coefficient) are temporally complex, situated between complete order and disorder. Our results also indicated that “segregated network nodes” may attempt to minimize the network’s entropy, whereas “integrated network nodes” require a higher information load, and therefore need to increase entropy. We believe that combining temporal information from functional brain networks and entropy can be used to test the decomplexification theory of disease, especially in neurological and psychiatric conditions characterized by paroxysmal brain abnormalities (e.g., schizophrenia and epilepsy).

The brain is complex. One of the most convincing examples of this is the neuron, with its lognormal firing rate and critical states (Chialvo, 2010). However, not much is known about the spatiotemporal complexity underlying large-scale brain networks. Nevertheless, it is feasible to estimate the complexity of brain networks in functional magnetic resonance imaging (fMRI) by using measures of signal entropy (Bassett, Nelson, Mueller, Camchong, & Lim, 2012). Entropy reveals the extent to which a signal is temporally ordered (low entropy), uncorrelated (high entropy), or complex (medium entropy). Entropy has been used in a variety of settings, with notable contributions to cardiovascular disease markers such as heart-rate variability (Lake, Richman, Griffin, & Moorman, 2002). The entropy of spontaneous brain activity signals has received increasing attention, and a few empirical studies have started to explore the entropy of fMRI signals in healthy people (McDonough & Nashiro, 2014; Wang, Li, Childress, & Detre, 2014) and in disease populations (Bassett et al., 2012; Sokunbi et al., 2014).

*Connectomics* is a relatively new field in which the structure and function of brain networks is studied (Sporns, Tononi, & Kötter, 2005). Several graph-theoretic measures exist that quantify network properties, including measures of node degree, betweenness centrality, clustering coefficient, modularity, participation coefficient, and efficiency (Rubinov & Sporns, 2010). Given this wealth of options, a reductionist approach is appropriate when selecting the network measures for a study. In the present work, we wanted to use measures that may reflect topologically “segregated” and “integrated” network activity. The clustering coefficient (the “cliquiness” of a node) and participation coefficient (the intermodular connectivity of a node) are graph-theoretic measures that quantify, respectively, brain network segregation and integration (Guimerà & Nunes Amaral, 2005; Watts & Strogatz, 1998).

To obtain a realistic characterization of the temporal evolution of brain networks, time-varying functional connectivity information has been collected using fMRI (Chang & Glover, 2010; Handwerker, Roopchansingh, Gonzalez-Castillo, & Bandettini, 2012; Zalesky, Fornito, Cocchi, Gollo, & Breakspear, 2014). Moment-to-moment changes that occur in the brain are challenging to capture with fMRI-based network measures, due to the low-frequency nature of the hemodynamic response function (Glover, 2011). A commonly used approach for this purpose is *sliding-window analysis*, in which correlations within narrow segments of fMRI data are estimated over time. A promising alternative to sliding-window analysis is *instantaneous phase synchrony* analysis, which considers concurrent functional relationships between brain regions at the same temporal resolution of fMRI time series (Omidvarnia et al., 2016; Ponce-Alvarez et al., 2015).

To examine the temporal complexity of brain network properties, we estimated the sample entropy (*SampEn*) of clustering-coefficient and participation-coefficient time series, which were derived from fMRI connectivity matrices using instantaneous phase synchrony. We showed that quantifying the entropy of brain network properties enables us to link the temporal complexity and topology of functional brain networks, which may be used to characterize (altered) brain networks in disease.

## RESULTS

### Participation Coefficient and Clustering Coefficient Are Inversely Related

**Figure 1.**
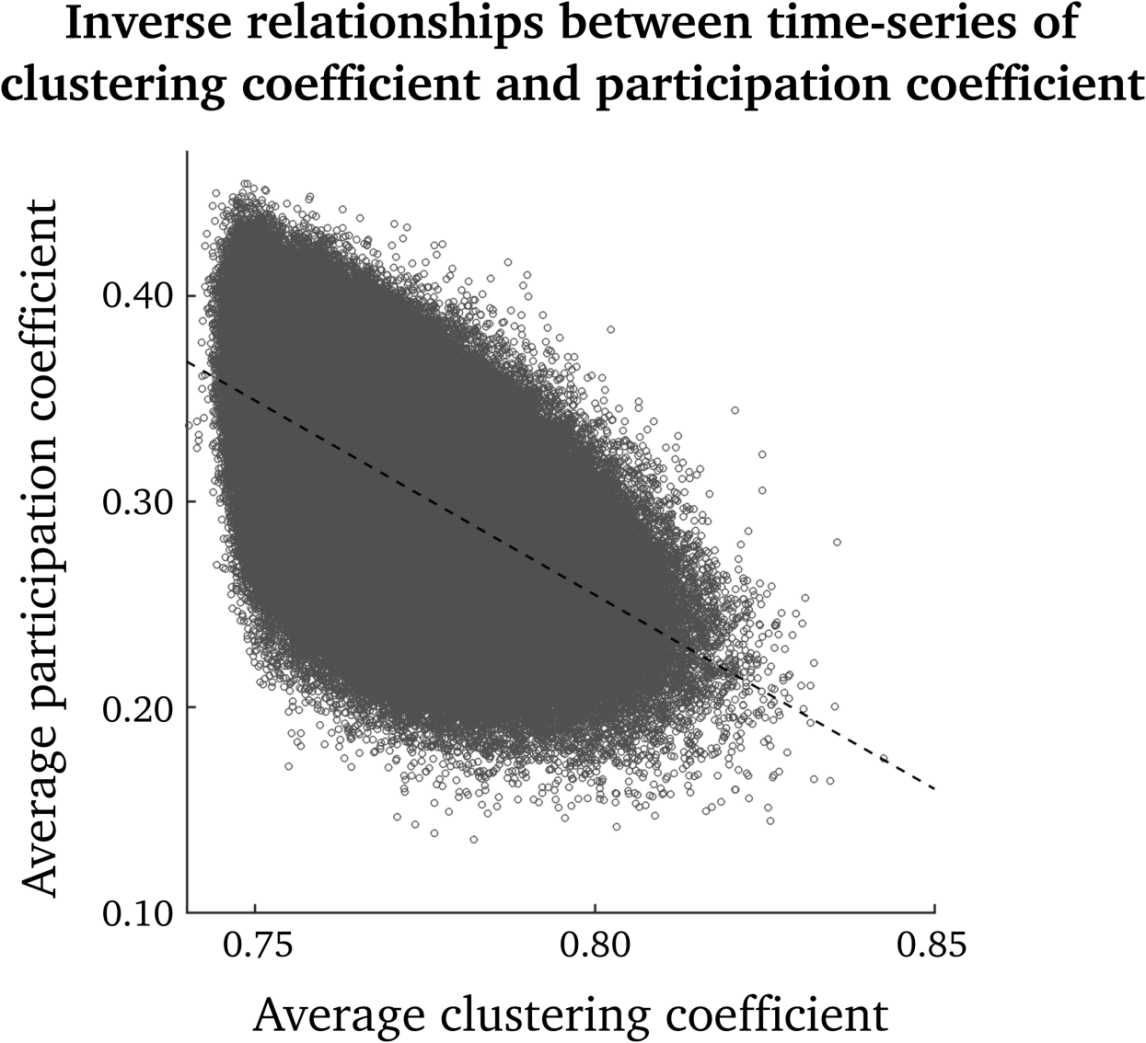
Scatterplot of participation-coefficient versus clustering-coefficient time series. Shown are all time points and nodes over the group of subjects. The dashed line corresponds to the best linear fit.

As can be seen in Figure 1, there is an inverse relationship between clustering-coefficient and participation-coefficient time series (i.e., time points with high clustering coefficients generally have low participation coefficients, and vice versa). Pearson’s correlation coefficient between the clustering coefficient and participation coefficient, pooled over all nodes and time points, was –0.56.

### SampEn Is Higher for the Participation Coefficient Than for the Clustering Coefficient

**Figure 2.**
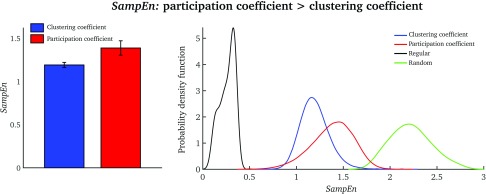
(Left) Average *SampEn* values over all nodes for the clustering coefficient and participation coefficient (a single value per subject). (Right) Node-wise *SampEn* distributions for all 25 subjects for the clustering coefficient (blue) and the participation coefficient (red). The regular distribution (black) was generated using sine waves of different frequencies, and the random distribution (green) was generated with MATLAB’s *rand* function (akin to the illustrative example seen in Figure 6). For the regular and random data, we generated signals equal in number and length to those in the fMRI data (blue and red).

*SampEn* was significantly higher for the participation coefficient than for the clustering coefficient (two-sample *t*-test = 11.06, *p* < 0.0001; see Figure 2, left). For both network measures, *SampEn* values were placed in-between completely regular and random time series (Figure 2, right).

### Relationships Between *SampEn* and the Participation Coefficient/Clustering Coefficient

**Figure 3.**
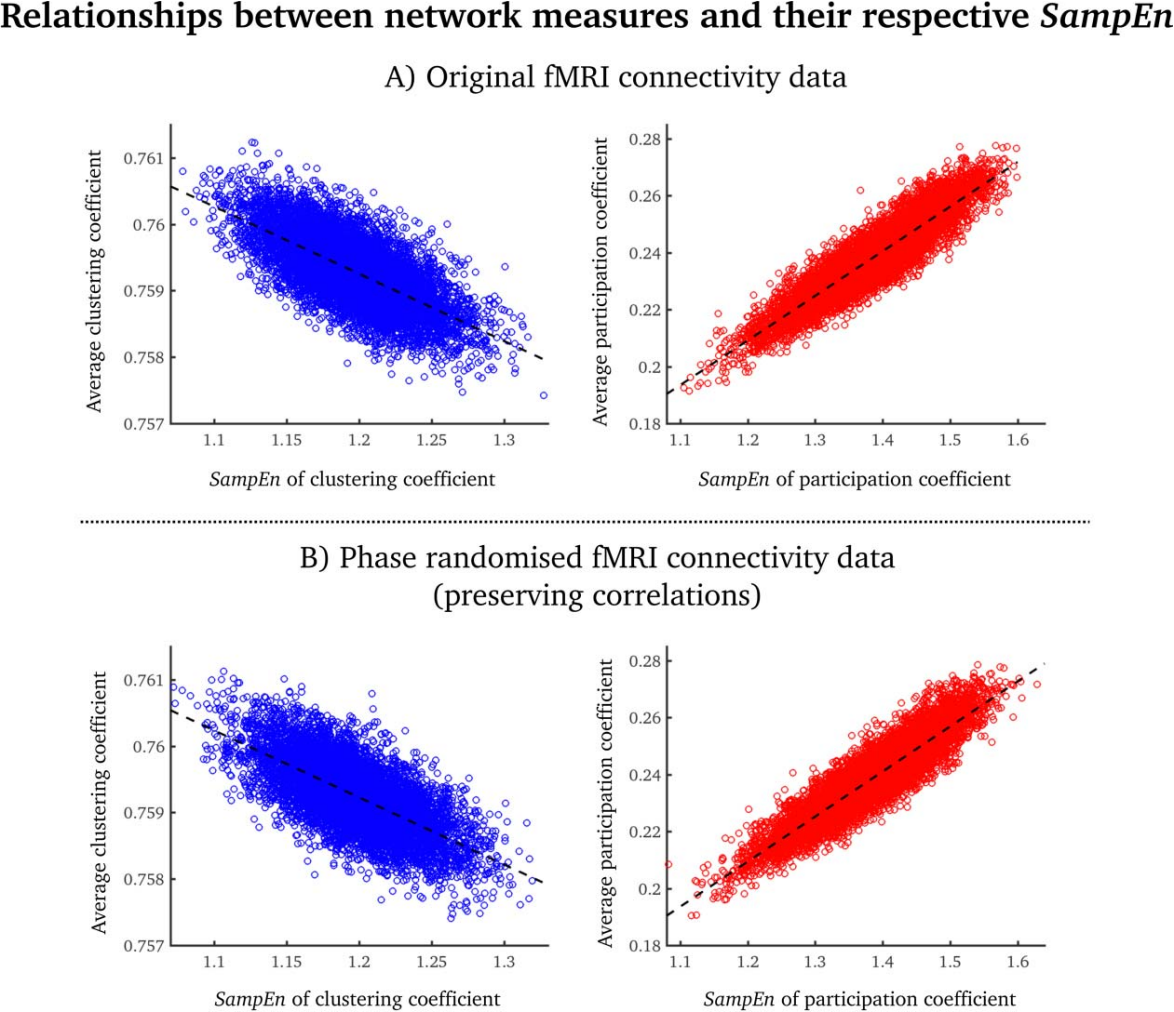
Scatterplots of average clustering coefficients (blue)/participation coefficients (red) and the *SampEn* of each network measure for the original fMRI data (A) and the phase-randomized fMRI data (B). Each point denotes a group-averaged node value. The dashed lines correspond to the best linear fit.

The average clustering coefficient was inversely related to the *SampEn* of clustering coefficient (Pearson’s correlation coefficient = –0.67; Figure 3A, left). The average participation coefficient was positively related to the *SampEn* of a participation coefficient (Pearson’s correlation coefficient = 0.90; Figure 3A, right). Near identical results were obtained from phase-randomized data where the correlation structure is preserved, and thus the relations between *SampEn* and the static measures are preserved. The relationship between *SampEn* and network activity are therefore likely to be due to the zero-lag correlation structure between nodes, rather than to nonstationarities.

### *SampEn* Is Only Network-Specific for the Participation Coefficient

**Figure 4.**
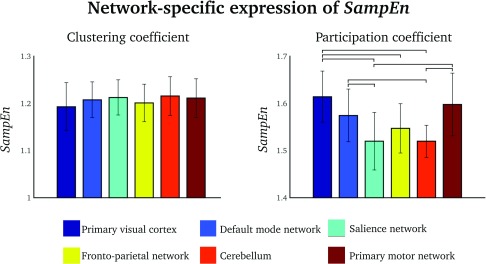
Group-level *SampEn* values of specific brain networks for the clustering coefficient (left) and the participation coefficient (right). Error bars = standard deviations. Lines = Bonferroni-corrected statistically significant pair-wise difference.

We evaluated the average *SampEn*s of the clustering coefficient and the participation coefficient within a number of functionally well-defined brain network nodes from the default mode network, salience network, frontoparietal network, primary sensory (visual and motor) networks, and cerebellum. These network nodes were defined using principal component analysis. This was done by calculating the first five principal components of the average group-level instantaneous phase synchrony data obtained in Step 3 in Materials and Methods (see Supplementary Information 1 (Pedersen et al., 2017) for more information). *SampEn* varied between specific network nodes for the participation coefficient (one-way ANOVA: *F* = 13.2, *p* < 0.0001), but not for the clustering coefficient (one-way ANOVA: *F* = 1.1, *p* = 0.39). Bonferroni-corrected post-hoc analysis revealed seven out of 15 significant comparisons, mostly in the primary visual cortex, default mode network, and primary motor network (see the paired differences in Figure 4, right).

### Replication Dataset: Human Connectome Project

To test whether our results were reproducible, we used task-free fMRI data from the Human Connectome Project (Van Essen et al., 2013). In this analysis we used a network parcellation scheme with fewer nodes (than in the analyses above), which allowed us to estimate *SampEn* over a range of network density thresholds.

**Figure 5.**
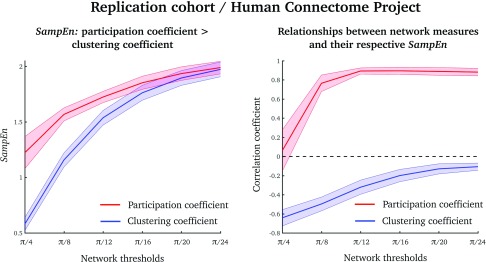
Results from a replication dataset over a range of network density thresholds (from *π*/4 to *π*/24). (Left) *SampEn* values of the participation coefficient (red) and clustering coefficient (blue), averaged over all nodes (akin to the results in Figure 2). (Right) Pearson’s correlation coefficients between the average clustering coefficient (blue) and participation coefficient (red) and their associated *SampEn*s (akin to the results in Figure 3). Means and standard deviations are displayed as lines and shaded colors, respectively.

The results from this replication dataset were similar to our original results. That is, participation coefficients had higher *SampEn* over a range of thresholds than did clustering coefficients (Figure 5, left). Also, the average clustering coefficient was negatively correlated with its *SampEn*, and the average participation coefficient was positive correlated with its *SampEn* (Figure 5, right).

## DISCUSSION

In this study, we combined functional connectivity, graph theory, and entropy to elucidate the temporal complexity of brain network properties. Although a few studies have previously measured complexity and *SampEn* on the basis of fMRI signals (e.g., Bassett et al., 2012; McDonough & Nashiro, 2014; Sokunbi et al., 2014; Wang et al., 2014), the present study was, to our knowledge, the first to directly assess the complexity of temporal fMRI network properties. We observed that the clustering-coefficient and participation-coefficient signals were more complex than regular, predictable systems (minimal entropy: see Figure 2, black distribution), but less uncertain than a random, uncorrelated system (maximal entropy: see Figure 2, green distribution). This is further evidence that human brain networks are situated between temporal order and disorder—that is, at a high level of complexity. The wider implications of the divergent relationship between the clustering coefficient and the participation coefficient, and their associated temporal complexity, will be discussed in the following sections.

### *SampEn*s of the Clustering Coefficient and the Participation Coefficient Are Differentially Expressed

The inverse relationship between the clustering coefficient and the participation coefficient suggests that the brain transits between network “segregation” and “integration.” This is in line with a recent study demonstrating that the brain switches between two distinct states of network segregation or integration (Shine et al., 2016).

Thus, the entropy of these processes may be of significance, since the “segregated” brain may attempt to minimize its own entropy—that is, nodes with high clustering coefficients display low *SampEn* (Figure 3, left). These particular nodes have a temporal pattern that is inclined toward temporal regularity, or predictability. This is consistent with existing theories of entropy in living systems. According to Erwin Schrödinger, any living system operates on the basis of *negentropy*—it will strive to minimize its own entropy (see *What Is Life: The Physical Aspect of the Living Cell*, published in 1944). In other words, living systems may need to be temporally ordered to function optimally in an otherwise chaotic world (Mahulikar & Herwig, 2009).

On the other hand, when the brain displays high “integration” (a high participation coefficient), *SampEn* is also high (Figure 3A, right). This finding may relate to the excessive information load imposed on these particular nodes when capturing between-module connectivity. This result resembles findings by Bassett et al. (2012), who found that wavelet entropy was positively related to node-wise fMRI strength (i.e., connectivity between a node and all other nodes in the network) in both healthy people and patients with schizophrenia. Using resting-state electroencephalogram recordings, Mišić, Vakorin, Paus, and McIntosh (2011) demonstrated that nodal measures of distributed connectivity (global efficiency, degree, and betweenness centrality) were positively correlated with *SampEn*. These studies reinforce the notion that the extent (or diversity) of network information may be related to the entropy, or unpredictability, of brain signals.

Network-specific entropy was a feature of participation-coefficient, but not of clustering-coefficient, time series (Figure 4). This finding implies that the participation coefficient is a metric that enables network-specific characterization. This is in line with the seminal work of Guimerà and Nunes Amaral (2005) on the participation coefficient. These authors demonstrated that in several network types, the clustering coefficient is not able to capture network-specific processes, but on the contrary, the participation coefficient was important for quantifying between-network connectivity in a range of networks (Guimerà & Nunes Amaral, 2005). In line with previous fMRI studies, we believe that the participation coefficient may be a unique and alternative measure of brain network activity (see Power, Schlaggar, Lessov-Schlaggar, & Petersen, 2013).

### On the Dynamics of fMRI Connectivity

Instantaneous phase synchrony is a relatively new way of deriving time-resolved connectivity using fMRI (see Glerean, Salmi, Lahnakoski, Jääskeläinen, & Sams, 2012; Omidvarnia et al., 2016; Ponce-Alvarez et al., 2015). Glerean et al. (2012) showed that instantaneous phase synchrony and correlation-based sliding window analysis detected comparable temporal properties. But they demonstrated that instantaneous phase synchrony was superior at achieving optimal temporal resolution (a single repetition time), especially since the reliability of correlation-based sliding windows decreases as the temporal window shrinks. This was also the case in our data. As can be seen in Supplementary Information 2 (Pedersen et al., 2017), we observed that instantaneous phase synchrony time series are similar to correlation-based sliding windows time series at short window lengths (<60 s).

The phase-randomized fMRI analysis that preserved the underlying correlational nature of our data suggests that the results were not predominantly driven by nonstationarities inherent in the data. This finding is concordant with Allen et al. (2014), who observed no changes in time-varying functional connectivity data after applying the same phase randomization procedure that we used (Prichard & Theiler, 1994). This ties in with a recent finding by Hindriks et al. (2016), who argued that dynamic fMRI connectivity methods may not detect nonstationarities in short resting-state scans of fMRI (∼ 10 min). Although the main point of the present study was to generate network time series appropriate for *SampEn* analysis, it will remain important for future studies to statistically evaluate the dynamic nature of fMRI data. Nevertheless, it is possible that our entropy findings signify persistent phase relationships between nodes that facilitate spontaneous brain network activity. It is also tempting to speculate that entropy may be partly constrained by the underlying structural network topology, given the significant role of network structure in shaping functional connectivity (Deco et al., 2013; Honey et al., 2009; Shen, Hutchison, Bezgin, Everling, & McIntosh, 2015).

### Why Should We Combine Functional Connectivity and Entropy?

Measures such as *SampEn* can be used to observe brain network changes that are associated with stereotypical paroxysmal diseases (e.g., schizophrenia and epilepsy). Regarding this point, Goldberger (1996) have proposed *decomplexification theory*, which posits that (nearly all) disease properties have an inherent tendency to be temporally ordered. These include (1) electrocardiograms during congestive heart failure, (2) autism and repetitive behaviors, (3) obsessions/compulsions, (4) Parkinsonian tremors, and (5) electroencephalograms of epileptic seizures (see Goldberger, 1996, for further information). This is in line with a recent study by Nedic et al. (2015), who demonstrated that patients with concurrent temporal-lobe epilepsy and hippocampal sclerosis show more temporally regular fMRI activity (estimated with an autocorrelation measure) proximate to the epileptogenic lesion. This finding reinforces that brain network nodes that are affected by disease may be temporally regular.

### Limitations

This study has several limitations. First, graph-theoretic fMRI results are dependent on their semi-arbitrary parameters. These parameters include the method of calculating of statistical dependence between nodes (e.g., correlation coefficients or phase synchrony), total number of nodes, thresholding, and binarization of the data. For example, introducing more or fewer connections in a graph (thereby increasing or decreasing the network density threshold) is likely to have profound effects on the interpretation of fMRI network measures (e.g., Zalesky et al., 2010). However, our replication dataset analysis suggests that our results are broadly consistent over a range of network density thresholds (Figure 5). Also, binarizing the data can result in the loss of valuable network information. In this study, we constructed large brain graphs to obtain rich spatial and temporal network information. Whether voxel-level (high spatial resolution) or node-level (low spatial resolution) brain networks are best remains a debated issue in fMRI (Stanley et al., 2013). Voxel-level networks avoid the need to define “biologically plausible” brain nodes and are robust against fragmentation. These high-resolution brain graphs are computationally demanding, however. Additionally, high-resolution brain graphs may contain “spurious” intracortical connections, although Hayasaka and Laurienti (2010) found that local connections had a minimal effect in sparse voxel-level graphs.

Second, we note that the dynamic modular decomposition we used will generate random community labels for each time point. But this has no bearing on calculation of the participation coefficient, which is a measure that is sensitive only to the spatial composition of modules, not to their (arbitrary) label. This is clearly demonstrated in our group-consensus partition of the Louvain community algorithm (see Supplementary Information 3) (Bassett et al., 2013; Betzel et al., 2013; Lancichinetti & Fortunato, 2012). Nodes that belong to the same module over time conform to well-known resting-state networks, including the default-mode network (see Supplementary Information 3 (Pedersen et al., 2017)). Further work may benefit from adopting the framework of Bassett et al. (2013; see also Mucha, Richardson, Macon, Porter, & Onnela 2010), who used time-resolved modular decompositions as a function of time, in which the decomposition at one time point was informed by previous time points.

Third, during time points with no internetwork connections, the participation coefficient returns a nodal value of 0, and the time-varying interpretation becomes sparse. Using simulations, we demonstrated that *SampEn* decreases when a signal becomes sparse (Supplementary Information 4 (Pedersen et al., 2017)). The *SampEn* of the participation coefficient (Figure 4, red) is therefore unlikely to be influenced by the inherent sparseness in this measure.

### Conclusion

By quantifying the entropy of dynamic brain networks, we were able to reconcile information about the temporal complexity and spatial topology of distinct brain network properties. We believe that combining network analysis and entropy may be useful to characterize dynamic brain networks in disease states.

## MATERIALS AND METHODS

### Step 1: fMRI Voxels Into Uniform Brain Parcellation

Using a uniform parcellation algorithm (Zalesky et al., 2010), we down-sampled the fMRI images (*N*_voxel_ = 51,603) associated with *T* = 200 volumes (**X** = *x*_*n*_ [*t*] ∈ ℝ^*N*_voxel_×*T*^, *n* = 1, … , *N*_voxel_; *t* = 1, … , *T*) into equal-sized brain nodes (*N*_node_ = 8,192) that included the cerebellum and subcortical structures of 25 healthy subjects. This resulted in a subject-specific 2-D matrix **Y** = *y*_*m*_ [*t*] ∈ ℝ^*N*_node_×*T*^ (*m* = 1, … , *N*_node_; *t* = 1, … , *T*). Each row of the matrix **Y** represents the average fMRI time series of a single node. For all 25 subjects, the pair-wise instantaneous phase coherence between nodes was estimated as we describe in the next section.

### Step 2: Instantaneous Phase Extraction

The pair-wise instantaneous phase coherence across nodes in **Y** was used as a time-varying measure of brain connectivity (Ponce-Alvarez et al., 2015). To this end, the concept of analytic signals based on the Hilbert transform (Mormann, Lehnertz, David, & Elger, 2000) was employed to extract the phase information of the mean fMRI time series in **Y**. Let *z*_*m*_1__ [*t*] and *z*_*m*_2__ [*t*] be the*analytic associates* of two rows in **Y**—that is *y*_*m*_1__ [*t*] and *y*_*m*_2__ [*t*]. In other words,

$$z_{m_{1}}\left[t\right]=y_{m_{1}}\left[t\right]+\tilde{jy_{m_{1}}}\left[t\right]=a_{m_{1}}\left[t\right]e^{j\varphi_{m_{1}}\left[t\right]}$$ (1)

$$z_{m_{2}}\left[t\right]=y_{m_{2}}\left[t\right]+\tilde{jy_{m_{2}}}\left[t\right]=a_{m_{2}}\left[t\right]e^{j\varphi_{m_{2}}\left[t\right]}$$ (2)

where $j=\sqrt{-1}$, the tilde symbols mean Hilbert transforms, *a*_*m*_1__ [*t*] and *a*_*m*_2__ [*t*] are instantaneous amplitudes, and the functions *φ*_*m*_1__ [*t*] and *φ*_*m*_2__ [*t*] represent the instantaneous phases of *y*_*m*_1__ [*t*] and *y*_*m*_2__ [*t*], respectively. The original signals are assumed to satisfy Bedrosian’s theorem—namely, the instantaneous amplitudes are slow-varying signals, and exponential terms are narrow-band signals having nonoverlapping spectra with instantaneous amplitudes (Bedrosian, 1963). The two signals *y*_*m*_1__ [*t*] and *y*_*m*_2__ [*t*] are said to be phase-locked of order 1:1 if

$$\left|\varphi_{m_{1}}\left[t\right]-\varphi_{m_{2}}\left[t\right]\right|\approx 0\text{.}$$ (3)

### Step 3: Instantaneous Phase Synchrony Matrices

The outcome of Step 2 for each subject-specific matrix **Y** is another same-size matrix **Z** = *z*_*m*_ [*t*] ∈ ℝ^*N*_node_×*T*^ (*m* = 1, … , *N*_node_; *t* = 1, … , *T*) including all analytic associates that have instantaneous phase information.

We quantified the instantaneous phase difference between the two phase signals *φ*_*m*_1__ [*t*] and *φ*_*m*_2__ [*t*] obtained from two typical rows of the analytic associate matrix **Z** as follows:

$$d_{m_{1},m_{2}}\left[t\right]=|\sin\;\left(\varphi_{m_{1}}\left[t\right]-\varphi_{m_{2}}\left[t\right]\right)|$$ (4)

The sine operator handles the phase-wrapping issue and removes the ambiguity of phase values over time. Repeating this procedure for all nodes and all time points results in a 3-D matrix **D** = *d*_*m*_1_,*m*_2__ [*t*] ∈ ℝ^*N*_node_×*N*_node_×*T*^ (*m*_1_,*m*_2_ = 1, … ; *N*_node_, *t* = 1, … , *T*) whose elements are restricted to the interval [0,1]. A value close to 1 reflects a high phase difference between *x* and *y* and a near-zero value implies a high level of phase synchrony. In other words, each time point of the 3-D matrix **D** is associated with a 2-D phase connectivity matrix representing all possible pair-wise phase comparisons among brain nodes. The matrix is symmetric in its first two dimensions.

### Step 4: Binary Thresholding of Instantaneous Phase Synchrony Matrices

Next, the instantaneous phase synchrony graphs were thresholded and binarized. Thresholding is a somewhat arbitrary, albeit necessary, step for interpreting brain graphs in a meaningful way (Langer, Pedroni, & Jäncke, 2013). There is currently no gold-standard approach for thresholding functional brain graphs (Achard & Bullmore, 2007). A reasonable way, however, would be to threshold graphs over multiple network densities covering a biologically plausible range of brain connections (see Pedersen, Omidvarnia, Walz, & Jackson, 2015, for an example). By a “network density,” we mean the proportion of connected nodes to all possible links in a network. A multiple-thresholding approach, on the other hand, is computationally demanding for large-size functional graphs with a high number of nodes. In this study, we thresholded and binarized the graphs such that edges were only drawn between nodes with a phase difference less than *π*/16. This was done to ensure that only high-phase-synchrony values between nodes would remain in the graphs. On average, 6.34% (±0.0018 standard deviation over subjects, ±0.0019 standard deviations over time points) of all possible edges were preserved in the thresholded connectivity matrices. The low variance at this threshold across both subjects and time points suggests that this threshold is stable. All networks displayed a small-world topology (average small-worldness = 1.92 ± 0.28 standard deviation; a network adheres to a small-world configuration at values >1) and is consistent with other studies combining graph theory and fMRI. Small-worldness was calculated by dividing the whole-brain averaged normalized clustering coefficient and the characteristic path length (across time points and subjects). A total of 500 random networks were calculated for these two normalized metrics. None of the resulting binary networks were fragmented, and the size of the largest subgraph (i.e., the *largest network component*) was equal to *N*_node_ = 8,192 for all subjects and time points.

### Step 5: Network Analysis and Null Model

To calculate network properties from the thresholded brain graphs, we used MATLAB-implemented functions from the Brain Connectivity Toolbox (www.brain-connectivity-toolbox.net/) and Boost Graph Library (https://www.cs.purdue.edu/homes/dgleich/packages/matlab_bgl/). We chose two biologically interpretable graph measures of brain connectivity—that is, the clustering coefficient and the participation coefficient.

The *clustering coefficient*, or *CC* (Watts & Strogatz, 1998), quantifies the proportion of neighboring nodes of a given node *i* that are clustered together. This measure is mathematically described for node *i* as

$$CC_{i}=\frac{2t_{i}}{k_{i}\left(k_{i}-1\right)}\text{,}$$ (5)

where *t*_*i*_ denotes the number of triangles surrounding node *i* and *k*_*i*_ is its degree (i.e., number of network-wide links connected to it). The values *CC*_*i*_ always range within [0, 1].

The *participation coefficient*, or *PC* (Guimerà & Nunes Amaral, 2005), quantifies the diversity of information between network modules. For node *i*, the metric is written as

$$PC_{i}=1-\sum_{m\in M}{\left(\frac{k_{i}\left(m\right)}{k_{i}}\right)}^{2}\text{,}$$ (6)

where the parameter *M* denotes a set of modules that subdivide the network into nonoverlapping partitions, and the parameter *k*_*i*_(*m*) counts the number of connections between node *i* and all nodes in module *m*. The participation coefficient always takes values in the range [0, 1]; 0 means that all nodal connections are either intramodular or intermodular to the same module. A value of 1 means that all nodal connections are intermodular (with connections to a variety of modules). In this study, *M* was calculated for each time point and subject, using a Louvain community structure algorithm with a community affiliation vector of *γ* = 2 (Blondel, Guillaume, Lambiotte, & Lefebvre, 2008). We chose *γ* = 2 in contrast to *γ* = 1 to allow for a finer spatial distinction between modules, since this reduces the probability of zero values for the participation coefficient (i.e., a node having only intranetwork connections). This clustering method contains heuristics that may cause run-to-run variability. To estimate the extent of this variability, we randomly selected 50 of the instantaneous phase synchrony matrices used in this study and calculated the average modularity of each (the *Q*-score) 200 times. The average *Q*-score over the runs was 0.59 with a standard deviation of 0.002 (coefficient of variance = 0.003). Thus, the run-to-run variability of the Louvain community structure algorithm used in this study appears to be low. The median number of modules across subjects was 6 (minimum number of modules = 1; maximum number of modules = 14).

Our main results were also compared to results generated from fMRI phase-randomized data in which the fMRI time series were phase-shuffled in the Fourier domain while preserving the power spectral magnitude and the correlational nature of the data (Prichard & Theiler, 1994). Thus, the only aspect we changed was the inherent dynamics of the original fMRI time series. This manipulation therefore tested whether the underlying fMRI connectivity data were likely to be nonstationary—that is, did the statistical distribution change over time?

### Step 6: Entropy Analysis in the Time Domain

**Figure 6.**
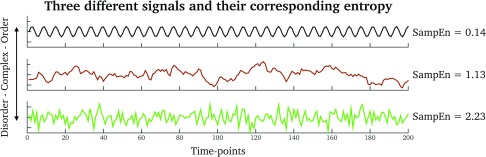
Examples of signals having different *SampEn* values. Top row (black signal): A regular signal. Middle row (brown signal): A fractal Brownian-motion signal. Bottom row (green signal): A random signal.

The last analysis step was devoted to the extraction of *SampEn* (Richman & Moorman, 2000) from the clustering-coefficient and participation-coefficient time series, from the original fMRI data as well as from the phase-randomized surrogates. *SampEn* is related to “the rate of generation of new information” in a signal. For example, periodic signals with high self-similarity will generate trivial “new” information by evolving in time (i.e., low *SampEn*), whereas biological signals with less self-similarity will have more information (i.e., high *SampEn*). Figure 6 illustrates three signals with different *SampEn* values. See also Supplementary Information 5 (Pedersen et al., 2017) for example time series of real fMRI clustering coefficients and participation coefficients.

Mathematically, *SampEn*(*m*, *r*, *N*) estimates the conditional probability that two templates of a signal will remain similar over time, when self-matches have been already excluded (see Richman & Moorman, 2000, and Sokunbi et al., 2013, for overviews). Here, the term “template” refers to *m*-dimensioanal vectors made by the delayed time points in the original signal. This is governed by four parameters: *N*, *m*, *τ*, and *r*, where *N* denotes the number of time points in the entire signal, *m* and *τ* specify the segmented and delayed templates in the original signal, and *r* is a threshold controlling the level of similarity between templates. The measure is defined as

$$\mathrm{SampEn}\left(m,r,N\right)=-\ln\left(\frac{U^{m+1}\left(r\right)}{U^{m}\left(r\right)}\right)\text{,}$$ (7)

where *ln* denotes the natural logarithm and *U*^*m*^(*r*) is defined as

$$U^{m}\left(r\right)=\frac{1}{N-m\tau }\sum_{i=1}^{N-m\tau }C_{i}^{m}\left(r\right)\text{.}$$ (8)

Equation (8) is based on the probability functions $C_{i}^{m}(r)$ formed by the average number of *m*-length templates *X*_*j*_ = [*x*_*j*_, *x*_*j* +*τ*_, … , *x*_*j* +(*m*−1)*τ*_](1 ≤ *j* ≤ *N* − *mτ*), which are closely similar to the template *X*_*i*_ (*i* ≠ *j*):

$$C_{i}^{m}\left(r\right)=\frac{B_{i}}{N-\left(m+1\right)\tau }\text{.}$$ (9)

The level of “similarity” is quantified by the value *B*_*i*_ as the number of templates *X*_*j*_ whose distance to the template *X*_*i*_ is less than or equal to *r*:

$$d\left|X_{i},X_{j}\right|\leq r\text{.}$$ (10)

The distance metric *d* between two time series is defined as the maximum difference between their corresponding time points—that is, $d\left|X_{i},X_{j}\right|=\max_{0\leq k<m-1}\left|X_{i+k}-X_{j+k}\right|$. In this study, the parameter *r* was set to 0.2 times the standard deviation of the original signal allowing for the comparison of signals with different amplitudes. This adaptive tolerance parameter is important because biological signals such as clustering coefficient and participation coefficient time series may change and fluctuate at different amplitude scalings. See Supplementary Information 6 (Pedersen et al., 2017) for illustrations of the amplitude changes and entropy. With the total length of *N* = 200 for the datasets of this study, we chose the parameters *m* and *τ* in *SampEn*(*m*, *r*, *N*) as 2 and 1, respectively. One of the advantages of *SampEn* is its reliability when estimating only a few time points. In Supplementary Information 7 (Pedersen et al., 2017), we use simulated data to demonstrate that 200 time points (the same length as our fMRI data) is sufficient to obtain stable *SampEn* values (see also Yentes et al., 2013). Although our *SampEn* parameters are in line with previous studies (McIntosh, Kovacevic, & Itier, 2008; Sokunbi et al., 2014), deciding upon optimal parameters for *SampEn* is still an open issue (Alcaraz, Abásolo, Hornero, & Rieta, 2010).

### fMRI Parameters and Preprocessing

The task-free fMRI data of 25 healthy control subjects (eyes closed) were included in our analysis (mean age = 24.6 years, ±3.6 standard deviation). The study was approved by the Austin Health Human Research Ethics Committee, Austin Hospital, Melbourne, Australia. All subjects gave written informed consent to participate in the study.

Data were recorded using a 3-Tesla Siemens Skyra MRI system (Erlanger, Germany) with 44 slices (3 mm thick), a repetition time (TR) of 3 s, an echo time of 30 ms, a flip angle of 85°, and a voxel size of 3 × 3 × 3 mm. In all, 10 min (200 time points) of fMRI data were obtained per subject. We used SPM12 (Friston, Ashburner, Kiebel, Nichols, & Penny, 2007) and DPABI (Yan, Wang, Zuo, & Zang, 2016) for preprocessing the data in MATLAB R2016a (MathWorks Inc., Natick, Massachusetts, US). The initial preprocessing steps included slice-time correction, realignment, and co-registration to the subject’s own T_1_-weighted space. Each fMRI volume was then segmented into separate tissue types (gray matter, white matter, and cerebrospinal fluid) using DARTEL (Ashburner, 2007). The average signal from the cerebrospinal fluid and white matter, as well as 24 motion parameters (Friston, Williams, Howard, Frackowiak, & Turner, 1996), was regressed out from the fMRI data.

Images were normalized into the Montreal Neurological Institute (MNI) space with an isotropic voxel size of 3 × 3 × 3 mm. The data were band-pass filtered within the frequency band of 0.03–0.07 Hz. This frequency interval satisfies Bedrosian’s theorem’s requirements for fMRI phase synchrony estimation, and it is also minimally affected by different artifacts including respiration (lower-frequency) and cardiac (higher-frequency) artifacts (Glerean et al., 2012; see also Omidvarnia et al., 2016, for a discussion on the Bedrosian requirement and fMRI phase synchrony). To counteract adverse effects due to excessive in-scanner head movement, all brain volumes associated with a frame-wise displacement of 0.5 mm or higher were excluded (Power, Barnes, Snyder, Schlaggar, & Petersen, 2012) and replaced using a cubic spline temporal interpolation. This interpolation procedure has been used in previous dynamic fMRI connectivity studies (Thompson & Fransson, 2015). An average of 3.9% (±3.5% standard deviation) of the fMRI time points were interpolated for each subject.

### Replication Dataset

We also analyzed the task-free fMRI data of ten healthy subjects (age range = 22–35 years) from the Human Connectome Project (Van Essen et al., 2013) (left–right encoded, first session, Q1 release) with 72 slices (2 mm thick), TR of 0.72 s, echo time of 58 ms, flip angle of 90°, and voxel size of 2 × 2 × 2 mm. In total, 14.4 min (1,200 time points) of multiband fMRI data were obtained per subject. We decided to download the unprocessed fMRI data (www.humanconnectome.org/) to ensure that these data were preprocessed in the same way as the previous analysis, but without slice-time correction.

We changed several parameters from the previous analysis, to determine the overall reproducibility of our network entropy approach. First, we used a coarser parcellation scheme, with 1,024 nodes. This allowed us to estimate *SampEn* over a range of network density thresholds We selected the following six thresholds with the average network density percentages presented in parentheses: *π*/4 (26%), *π*/8 (13%), *π*/12 (9%), *π*/16 (7%), *π*/20 (5%), and *π*/24 (4%). As compared to the original analysis, we set the Louvain modularity parameter to *γ* = 1 instead of *γ* = 2. This was done to test whether this parameter influenced our results. We noted that the combination of this modularity parameter with the coarser parcellation scheme introduced more sparsity in the participation-coefficient time series. To avoid interpreting spuriously low *SampEn* values in the participation-coefficient time series, we calculated *SampEn* for nonzero data only (see Supplementary Information 4 (Pedersen et al., 2017) for an explanation of this issue). This was feasible because of the high degrees of freedom present in this data (the total number of time points per subject was 1,200).

## ACKNOWLEDGMENTS

Data were provided by the Human Connectome Project, WU–Minn Consortium (1U54MH091657; principal investigators David Van Essen and Kamil Ugurbil), funded by the 16 National Institutes of Health (NIH) institutes and centers that support the NIH Blueprint for Neuroscience Research; and by the McDonnell Center for Systems Neuroscience at Washington University. We thank Mira Semmelroch, Donna Parker, and Magdalena Kowalczyk for assistance with the fMRI data acquisition. This study was supported by the National Health and Medical Research Council (NHMRC) of Australia (#628952). The Florey Institute of Neuroscience and Mental Health acknowledges strong support from the Victorian Government, and in particular, funding from the Operational Infrastructure Support Grant. We also acknowledge the facilities and the scientific and technical assistance of the National Imaging Facility at the Florey node. G.J. is supported by an NHMRC practitioner’s fellowship (#1060312) and has received honoraria from UCB and royalties from Elsevier for *Magnetic Resonance in Epilepsy*, 2nd edition.

## TECHNICAL TERMS

Functional magnetic resonance imaging (fMRI): An imaging technique capturing hemodynamic interactions in the brain with millimeter resolution.
Graph theory: A mathematical research field aiming to quantify the topological aspects of networks.
Clustering coefficient: Estimate the proportion of connected triangles surrounding a node; a high clustering coefficient is an indicator of network segregation.
Module: A collection of segregated nodes in the brain thought to subserve distinct functions.
Participation coefficient: Estimate of how well-connected the nodes within a given module are to other brain-wide modules; a measure of intermodular diversity.
Instantaneous phase synchrony: A measure that quantifies the functional relationships between brain nodes at each fMRI time point.
Sample entropy (*SampEn*): The rate of generation of new information in a signal; a measure of signal complexity.
Decomplexification theory: The theory that specific aspects of disease cause the brain to lose its “normal entropy”.
